# Monte Carlo simulations of EBT3 film dose deposition for percentage depth dose (PDD) curve evaluation

**DOI:** 10.1002/acm2.13078

**Published:** 2020-11-06

**Authors:** Spencer M. Robinson, Nolan Esplen, Derek Wells, Magdalena Bazalova‐Carter

**Affiliations:** ^1^ Department of Physics and Astronomy University of Victoria Victoria BC Canada; ^2^ BC Cancer Vancouver Island Centre Victoria BC Canada

**Keywords:** beam orientation, dosimetry, films

## Abstract

**Purpose:**

To use Monte Carlo (MC) calculations to evaluate the effects of Gafchromic EBT3 film orientation on percentage depth dose (PDD) curves.

**Methods:**

Dose deposition in films placed in a water phantom, and oriented either parallel or perpendicular with respect to beam axis, were simulated with MC and compared to PDDs scored in a homogenous water phantom. The effects of introducing 0.01–1.00 mm air gaps on each side of the film as well as a small 1°‐3° tilt for film placed in parallel orientation were studied. PDDs scored based on two published EBT3 film compositions were compared. Three photon beam energies of 120 kVp, 220 kVp, and 6 MV and three field sizes between 1 × 1 and 5 × 5 cm^2^ were considered. Experimental PDDs for a 6‐MV 3 × 3 cm^2^ beam were acquired.

**Results:**

PDD curves for films in perpendicular orientation more closely agreed to water PDDs than films placed in parallel orientation. The maximum difference between film and water PDD for films in parallel orientation was −12.9% for the 220 kVp beam. For the perpendicular film orientation, the maximum difference decreased to 5.7% for the 120 kVp beam. The inclusion of an air gap had the largest effect on the 6‐MV 1 × 1 cm^2^ beam, for which the dose in the buildup region was underestimated by 21.2% compared to the simulation with no air gap. A 2° film tilt decreased the difference between the parallel film and homogeneous water phantom PDDs from −5.0% to −0.5% for the 6 MV 3 × 3 cm^2^ beam. The “newer” EBT3 film composition resulted in larger PDD discrepancies than the previous composition. Experimental film data qualitatively agreed with MC simulations.

**Conclusions:**

PDD measurements with films should either be performed with film in perpendicular orientation to the beam axis or in parallel orientation with a ~ 2º tilt and no air gaps.

## 1 Introduction

Dose measurements using radiochromic film have become increasingly routine in radiation therapy and imaging.^1^ To date, film dosimetry has been used in a variety of therapeutic applications, including dose verification on the CyberKnife,^2^ in anthropomorphic breast phantoms^3^ and for intensity‐modulated radiotherapy.^4^ Other useful applications have included the evaluation of micro‐multileaf collimators,^5^ microbeam therapy,^6^ and *in vivo* dosimetry for total body^7^ and total electron skin^8^ irradiations. Films also find use in particle beams, including for proton^9^ or electron^10^ beam dosimetry and are considered for the use in MR‐guided radiotherapy.^11^ Radiochromic films have also been used for diagnostic applications, such as for computed tomography dose measurements.^12^, ^13^ An important advantage of film dosimetry over other dosimetry techniques is its high spatial resolution, 2D measurement capabilities, and low‐energy dependence.^14^ In this work, we focus on the investigation of percentage depth dose (PDD) curves derived from using radiochromic films in a variety of commonly used configurations.

The PDD is an important metric of interest in radiation therapy. To avoid conducting time‐consuming measurements using an ionization chamber in a water tank, PDDs are often measured with radiochromic films placed in a solid water phantom. This is typically achieved with two phantom setups. In the first configuration, a number of film sheets are sandwiched in a phantom in perpendicular orientation with respect to the beam axis and a small number of points for PDD evaluation corresponding to the number of sheets can be obtained.^15^, ^16^, ^17^, ^18^ Alternatively, a single sheet of film can be sandwiched in a phantom in parallel orientation with respect to the beam axis, which results in a practically continuous quantification of the PDD curve.^19^, ^20^, ^21^ In the present work, the potential differences in accuracy of PDD measurements between these two film phantom setups have been evaluated primarily by means of computer simulation.

Investigations of radiographic film orientation for electron beam isodose distributions date back to the late 1960s.^22^ Later in 1981, Williamson et al. investigated how film optical density (OD) changed as a function of film orientation and depth in phantom.^23^ The study revealed that radiographic (Kodak V2) film aligned parallel to the beam axis was more sensitive at higher depths compared to the film aligned perpendicularly. The authors developed a stoichiometric procedure to correct for the varying film energy response at depth. In 1999, Suchowerska et al. used experiments and Monte Carlo (MC) simulations to demonstrate that PDD measurements performed using radiographic films (Kodak X Omat‐V) in perpendicular orientation matched PDD measurements taken with an ionization chamber.^24^ In their study, the authors found that film in parallel orientation over‐responded by as much as 14% for a 6‐MV beam and an unspecified field size, which was in agreement with the results presented in the study by Williamson et al. The effect of film orientation on dose distributions has also been investigated more recently. In a 2001 follow‐up study, Suchowerska et al. found that the first generation Gafchromic^TM^ film over‐responded when placed in the parallel orientation compared to perpendicular orientation.^25^ They recommended that a gantry tilt of at least 2° should be introduced for accurate dose measurement with films in parallel orientation.

The main goal of this work was to use MC simulations to investigate the effect of Gafchromic^TM^ EBT3 film (ISP, Wayne, NJ) orientation (parallel and perpendicular to beam axis) on PDDs for a number of field sizes and beam energies. For films placed parallel to the beam axis, the effect of a slight 1‐3° film tilt and air gaps, which can exist between the film and the phantom, on PDDs was also evaluated.

## 2 Method

### 2.A Phantom and films

**Fig. 1:**
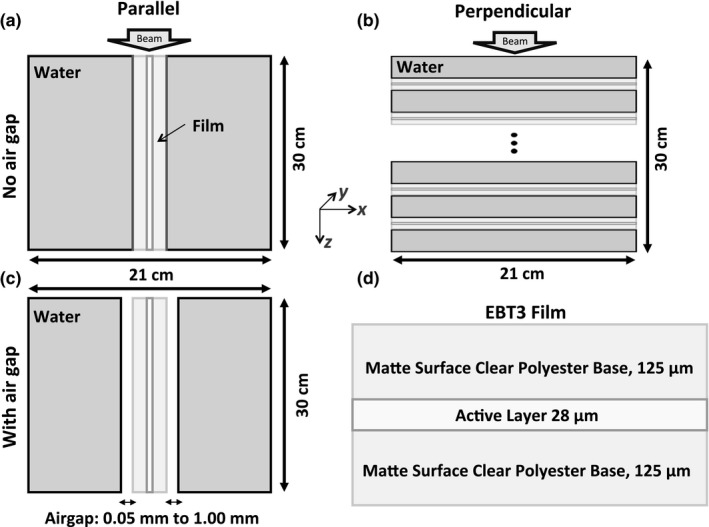
Simulation setup of phantoms with films in parallel (a) and perpendicular (b) orientation with respect to the beam axis. Air gaps were introduced in the phantom with a film parallel to beam axis (c). The geometry of Gafchromic EBT3 films (d).

To identify and quantify the effect that film orientation has on PDDs, two film orientations inside a 21 × 21 × 30 cm^3^ water phantom were considered: a single film parallel to the beam axis and a series of films perpendicular to the beam axis (**Fig.** **1**). One film was placed in the center of the phantom and aligned with the beam axis for the parallel film orientation setup [Fig. 1(a)]. For the perpendicular film orientation setup, 29 films were simultaneously placed at depths of 1‐29 cm in 1‐cm increments [Fig. 1(b)]. The effect of introducing a slight tilt or an air gap in the water phantom for film placed in parallel orientation was also evaluated. The film tilt was modeled by changing the beam incidence angle by 1–3° and the air gap effect was investigated by introducing air gaps of 0.05–1 mm on both sides of the film [Fig. 1(c)]. As a reference, dose distributions in a 21 × 21 × 30 cm^3^ homogeneous water phantom were also evaluated. The geometry of Gafchromic^TM^ EBT3 film studied in this work is depicted in [Fig. 1(d)]. Two EBT3 film compositions obtained from past studies were considered^26^, ^27^ and are listed in Table 1. The composition by Palmer et al., which contains 1.6% of aluminum in the active layer, is considered the “newer” one and it is used in all simulations unless stated otherwise.

**Table 1:** Two types of Gafchromic**^TM^** EBT3 film composition considered in this work. Data are from Palmer et al.^26^ and Bekerat et al. ^27^

EBT3 film	Density (g/cm^3^)	Composition (weight percentage)									
		H	Li	C	N	O	Na	S	Cl	Br	Al
Palmer et al.											
Polyester base	1.35	36.4	…	45.5	…	18.2	…	…	…	…	…
Active layer	1.20	56.8	0.6	27.6	…	13.3	…	…	…	…	1.6
Bekerat et al.											
Polyester base	1.35	4.0	…	63.0	…	33.0	…	…	…	…	…
Active layer	1.20	9.7	0.9	58.4	0.1	28.4	0.4	0.2	1.1	0.8	…

Three photon beam energies of 120 kVp, 220 kVp, and 6 MV and three field sizes of 1 × 1 cm^2^, 3 × 3 cm^2^, and 5 × 5 cm^2^ were considered in our study. The kilovoltage 120 kVp, the orthovoltage 220 kVp, and the high‐energy 6‐MV photon beam energies were chosen to represent typical imaging, small animal radiotherapy, and clinical radiotherapy beams, respectively.

### 2.B Monte Carlo simulations

All MC dose calculations were performed in the EGSnrc/DOSXYZnrc MC code.^28^

#### 2.B.1 Phantoms

Voxelized phantoms consisting of water and Gafchromic^TM^ EBT3 films were generated using MATLAB (version R2018b, The Mathworks, Nattick, MA). Each phantom was modeled as a 21 × 21 × 30 cm^3^ rectangular volume [Fig. 1(a)‐1(c)] and all films were simulated with the appropriate dimensions and composition as shown in [Fig. 1(d)] and listed in Table 1. Unless stated otherwise, the film composition published by Palmer et al.^26^ was used.

The phantom with films in parallel orientation [Fig. 1(a)] consisted of 109, 105, 150 voxels, in *x‐*, *y‐,* and *z‐*directions, respectively. In the *x*‐direction, the voxel sizes were 0.2 cm in water, 0.0125 cm in the EBT3 polyester layer, and 0.0028 cm for EBT3 active layer, which was centered on the *x*‐axis. The two outermost edge voxels in the *x*‐direction were 0.0861 cm to achieve a total phantom length of 21 cm. The voxel size in the *y‐* and *z‐*directions was 0.2 cm for this phantom.

The phantom with films placed in perpendicular orientation [Fig. 1(b)] consisted of 105, 105, and 117 voxels in *x‐*, *y‐* and *z*‐directions, respectively. The voxel size in the *z*‐direction was 0.9722, 0.0125, and 0.0028 cm for the water, EBT3 polyester and active layers, respectively. Film active layers were centered at measurement depths of 1–29 cm in 1‐cm intervals. A uniform voxel size of 0.2 cm was simulated in the *x‐* and *y*‐directions.

In order to investigate the effect of air gaps on PDD, a separate phantom with air on either side of the film layer was simulated. Air voxels of 0.05, 0.10, 0.50, or 1.00 mm length along the *x*‐direction were introduced between the water and polyester layer in the phantom with the film in parallel orientation [Fig. 1(c)]. As a result, a phantom with 111, 105, and 150 voxels, in the *x‐*, *y‐,* and *z*‐directions, respectively, was simulated. The reference (homogeneous) water phantom without films consisted of 105 × 105 × 150 voxels with uniform 0.2 × 0.2 × 0.2 cm^3^ voxels.

#### 2.B.3 Beams

Three photon beam energies of 120 kV, 220 kV, and 6 MV were simulated. The energy spectra were generated by MC based on validated models of a 120‐kV microCT imaging beam^29^ and a small animal radiotherapy beam.^18^ The 6‐MV beam was simulated with the default DOSXYZnrc mohan6 spectrum.^30^ In most cases, the beam was incident at 0°, as shown in Fig. 1. Parallel rectangular beams with field sizes of 1 × 1 cm^2^, 3 × 3 cm^2^, and 5 × 5 cm^2^ were simulated using ISOURCE = 12 for all three beam energies and in each of the phantoms. The effect of EBT3 film elemental composition was only studied for the 220‐kVp and 6‐MV 1 × 1 cm^2^ beams. For the 1–3° film tilt study, the beam was rotated by 1–3° with respect to the *z*‐axis to simulate film tilt. The film tilt was studied for the 220‐kVp and 6‐MV 3 × 3 cm^2^ beams.

#### 2.B.4 Simulation setup

All relevant processes for high‐ and low‐energy photon and electron transport, such as Rayleigh scattering, electron‐impact ionization and pair production were included in the simulations and the XCOM cross‐section data were used. The electron and photon cutoff kinetic energies were 5 keV and no variance reduction techniques were used. For simulations of phantoms with films, the dose to the film active layer was reported. For the water phantom simulation, the dose to water was reported. The number of simulated histories were 7 × 10^8^, 7 × 10^9^, and 1.95 × 10^10^ for the 1 × 1 cm^2^, 3 × 3 cm^2^ and 5 × 5 cm^2^ field sizes, respectively, in order to achieve a central‐axis dose uncertainty of < 1%. The simulations were run in parallel on a 64‐bit Linux computer with 64 AMD Opteron 6738 cores and took between ~ 80 and ~ 1700 CPU hours to run, depending on the beam energy and field size.

### 2.C Experiments

Experimental verification of the simulated depth dose distributions was accomplished using a combination of film and relative ionization chamber measurements. All measurements were taken with a 6‐MV photon beam delivered by a Varian TrueBeam ® STx (Varian, Palo Alto, CA, USA) linear accelerator (linac).

In total, four film measurement sets (parallel, parallel 2° tilt, parallel 1‐mm gap, perpendicular) and one ionization chamber set were taken to evaluate the depth doses within a 30‐cm‐thick solid water phantom (RMI Gammex, Middleton, WI, USA) composed of 30 cm × 30 cm slabs of varying thickness. For each measurement set, 400 MU was delivered while using a 3 × 3 cm^2^ field size at an SSD of 100 cm.

Parallel measurement films were each cut into 5 × 25 cm^2^ strips and individually placed at the center of the 30‐cm tall stack of solid water — one film for each parallel measurement set. The linac gantry was then rotated to 90° to allow for irradiation along a beam axis parallel to the film plane. For the parallel 1‐mm gap measurements, stacks of three 1 cm × 1 cm EBT3 films were additionally placed at each corner of the phantom to create an air gap along the entire film plane, which was measured to be approximately 0.9 mm. The 2° tilt configuration involved an identical setup without the air gap, but with the gantry tilted relative to the film plane (gantry angle: 92°). In contrast with the parallel sets, perpendicular measurement films were cut into 5 × 5 cm^2^ pieces and placed simultaneously at ten different depths between 0.5 and 25 cm, in 0.5 to 5 cm intervals; in this case, the linac gantry was positioned at 0° such that the beam axis was perpendicular to the film plane.

Prior to film measurements, a batch of EBT3 Gafchromic film was calibrated for use in the clinical 6‐MV beam. The output of the linac at the time of the measurements was 0.99 cGy/monitor unit (MU) under standardized setup conditions (10 × 10 cm^2^ field size at an SSD of 98.5 cm and depth of 1.5 cm). Films in this calibration batch were cut into 2 cm × 2 cm pieces and handled according to the manufacturer's specifications^31^ and the AAPM TG‐55 report.^32^ A dose calibration curve was established by irradiating ten films in a perpendicular orientation to doses ranging from ~ 20 to 600 cGy.

All calibration and measurement films were scanned 24 h after exposure on an EPSON® 10000XL flatbed scanner (Epson America, Long Beach, CA, USA) at a resolution of 200 dpi. Since the prescribed doses remained below 8 Gy, the red channel response was selected for calculating the absorbed dose to water. To enable comparison against the MC‐derived depth doses, relative film doses have been presented for which a dose normalization point nearest to the depth of maximum dose d_max_ (~1.6 cm) has been selected so as to be consistent with the MC data normalization at 6 MV.

To complement the film data, relative ionization chamber measurements were taken using a PTW Pinpoint chamber (PTW, Freiburg, Germany) together with a PTW UNIDOS E Electrometer and a −300 V bias applied. The chamber was positioned at the center of the 3x3cm^2^ beam and irradiated at various effective depths between 0.53 and 23.53 cm, in 0.5 to 5 cm intervals, within the same solid water phantom described previously; the raw electrometer reading was then taken and normalized to provide the relative depth dose data. The raw chamber response data were normalized to the dose at a depth of 1.6 cm, interpolated from the point nearest to d_max_, thereby providing a secondary reference measurement for comparison with the normalized film data.

### 2.D Data analysis

The MC 3D dose distributions were analyzed in MATLAB and central axis PDDs were plotted. Unless stated otherwise, 120 kVp and 220 kVp PDD curves were normalized to dose scored at 1‐cm depth while 6‐MV PDD curves were normalized to dose scored at d_max_ = 1.6 cm. In the air gap study, all PDD curves were normalized to the maximum central axis dose calculated in the simulation with no air gap. The error bars on MC‐derived PDD curves represent the MC statistical uncertainty σ. The error bars on PDD difference curves *σ_diff_* comparing PDD1 and PDD2 have been calculated by $\sigma_{\mathrm{diff}}=\sqrt{\sigma_{PDD1}^{2}+\sigma_{PDD2}^{2}}$.

## 3 Results

**Fig. 2:**
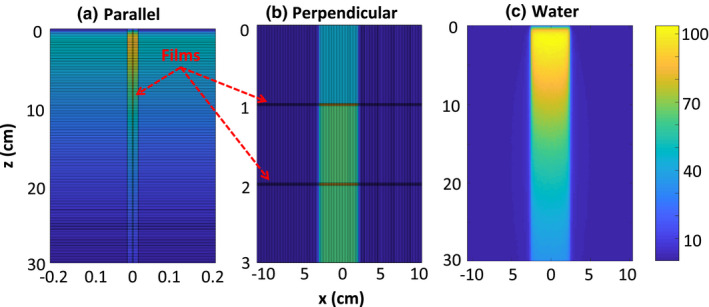
Normalized 2D dose distribution scored in the phantom with films in parallel (a), and perpendicular (b) orientation with respect to the beam axis, as well as in the water phantom (c) irradiated with the 6‐MV 5 × 5 cm^2^ beam. Doses are normalized to central axis dose scored at 1.6‐cm depth. Note the different scaling of the *z‐* and *x*‐axes.

Sample normalized 2D dose distributions for a 6‐MV 5 × 5 cm^2^ beam in the homogeneous water phantom and the phantoms with film oriented parallel and perpendicular to the beam axis are shown in Fig. 2. Comparisons of PDD curves for the two different film orientations as a function of field size and beam energy, as well as a function of air gap size for the parallel film orientation, are discussed below. Note that [Fig. 2(a)] indicates that the dose to water is lower than the dose to the EBT3 film polyester and active layers. This is due to the difference in mass energy‐absorption coefficients *µ_en_/ρ* of these two materials: at 2 MeV, the ratio of µ_en_/*ρ*of polyester to water is 1.22, which corresponds to the 1.25 dose ratio of the film polyester layer to water at 1.6‐cm depth.

### 3.A The effect of film orientation on PDD

**Fig. 3:**
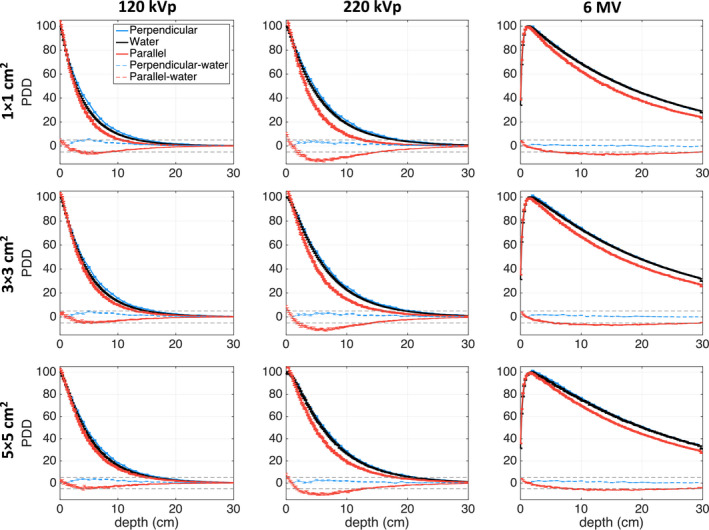
PDD plot comparison between parallel and perpendicular EBT3 film orientations in a water phantom and PDDs simulated for a water phantom for all three studied beam energies and field sizes. The dose difference line of ± 5% is indicated.

Figure 3 presents PDD curves for the water phantom and phantoms with film in parallel and perpendicular orientation for the three studied photon beam energies and field sizes. The maximum differences between the PDDs for the water phantom and those calculated for phantoms with films in parallel and perpendicular orientation are summarized in Table 2.

**Table 2:** Maximum differences between PDDs calculated for the parallel or perpendicular film orientation phantoms and thewater phantom PDD (film‐water) for all three beam energies and field sizes.

	120 kVp			220 kVp			6 MV		
	1 × 1 cm^2^	3 × 3 cm^2^	5 × 5 cm2	1 × 1 cm^2^	3 × 3 cm^2^	5 × 5 cm^2^	1 × 1 cm^2^	3 × 3 cm^2^	5 × 5 cm^2^
Parallel	−6.6%	−5.2%	−5.9%	−12.9%	−11.4%	−10.6%	−7.7%	−7.2%	−6.6%
	4.8 cm	5.4 cm	4.0 cm	6.2 cm	6.6 cm	6.2 cm	13.6 cm	15.0 cm	11.2 cm
Perpendicular	5.7%	4.7%	3.8%	3.7%	3.3%	2.7%	1.4%	2.0%	2.3%
	5.0 cm	5.0 cm	6.0 cm	4.0 cm	7.0 cm	5.0 cm	2.0 cm	7.0 cm	6.0 cm

For all beam energies and field sizes, the parallel film orientation resulted in poorer agreement with the water phantom simulations. Moreover, smaller field sizes generally resulted in larger dose discrepancies. For the parallel film simulations, the dose differences were largest for the 220‐kV beam, up to −12.9% at 6.2‐cm depth for the 1 × 1 cm^2^ field size. Dose with film in parallel orientation was underestimated by −7.7% at 13.6‐cm depth for the 6‐MV 1 × 1 cm^2^ beam. For the film in perpendicular orientation, increasing the beam energy resulted in a better agreement with the water phantom PDD, where the largest difference was found to be −5.7% at 5‐cm depth for the 120‐kVp 1 × 1 cm^2^ beam.

### 3.B The effect of air gap on PDD

**Fig. 4:**
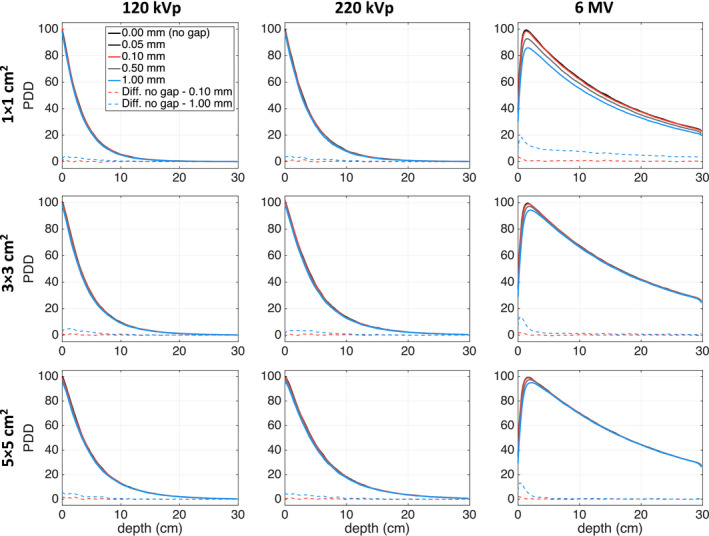
PDD curves for film in parallel orientation as a function on air gap size. Statistical uncertainty of all simulations is < 1%.

Parallel orientation film PDDs with varying air gaps for all beam energies and field sizes are plotted in Fig. 4. In most cases, the dose difference relative to a PDD with no air gap increased with increasing air gap size and decreasing field size.

Figure 4 clearly demonstrates this trend for the 6‐MV beams, for which the largest discrepancies occur in the build‐up region. The maximum PDD differences between the simulation without an air gap and those with 0.10‐mm and 1.00‐mm air gaps are summarized in Table 3. The largest dose discrepancy occurred for the 6‐MV 1 × 1 cm^2^ photon beam, for which the dose at 0.4‐cm depth was underestimated by 21.2% in the 1‐mm air gap simulation. In general, the largest dose discrepancies occurred at shallow depths and, with the exception of the smallest 6‐MV 1 × 1 cm^2^ photon beam, all dose differences were <3% for measurement depths >3 cm. Dose differences >7.5% were still found at depths of >10 cm for the 6‐MV 1 × 1 cm^2^ photon beam.

**Table 3:** Maximum differences between PDDs calculated for a phantom with film in parallel orientation without an air gap and with a 0.10‐mm or 1.00‐mm air gap (no air gap–air gap) for all three beam energies and field sizes.

Air gap	120 kVp			220 kVp			6 MV		
	1 × 1 cm^2^	3 × 3 cm^2^	5 × 5 cm^2^	1 × 1 cm^2^	3 × 3 cm^2^	5 × 5 cm^2^	1 × 1 cm^2^	3 × 3 cm^2^	5 × 5 cm^2^
0.10 mm	2.8%	1.9%	2.3%	2.4%	2.3%	2.1%	4.0%	2.5%	3.0%
	0.2 cm	2.2 cm	0.4 cm	2.8 cm	0.6 cm	0.8 cm	0.4 cm	0.2 cm	0.2 cm
1.00 mm	4.4%	6.0%	5.7%	5.5%	4.4%	5.8%	21.2%	16.8%	16.2%
	1.2 cm	1.2 cm	0.0cm	0.8 cm	1.8 cm	0.4 cm	0.4 cm	0.2 cm	0.2 cm

### 3.C Film elemental composition

**Fig. 5:**
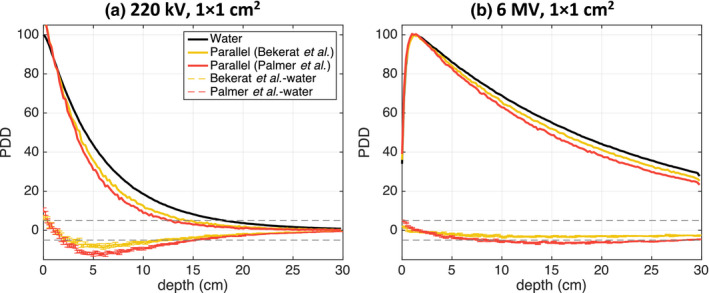
PDD curve comparison for films oriented parallel to the beam axis for two different compositions of Gafchromic EBT3 films for the 220‐kV 1 × 1 cm^2^ (a) and 6‐MV 1 × 1 cm^2^ beam (b). The dose difference line of ± 5% is indicated.

The results of investigating dose distribution differences for two different EBT3 film compositions by Palmer et al.^26^ and Bekerat et al.^27^ for the 220‐kVp and 6‐MV 1 × 1 cm^2^ beams are presented in Fig. 5. When comparing to the homogeneous water phantom PDD, the film composition from the study by Bekerat et al. resulted in more accurate dose calculations for a parallel film orientation than the “newer” composition presented by Palmer et al. For the Bekerat et al. composition, the maximum dose difference for the 220‐kVp and 6‐MV beams was −9.2% at 6.0‐cm depth and −3.3% at 10.2‐cm depth, respectively. In comparison, the maximum dose difference for the composition from Palmer et al. was higher at −12.9% at 6.2‐cm depth and −7.7% at 13.6‐cm depth for the 220‐kVp and 6‐MV beams, respectively.

### 3.D Film tilt

**Fig. 6:**
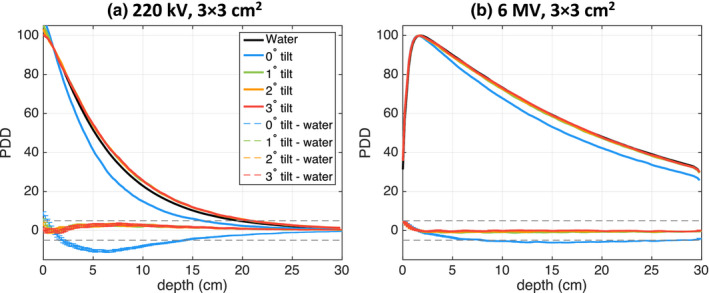
PDD curve comparison for films oriented at a slight tilt with respect to the beam axis for the 220‐kV 3 × 3 cm^2^ (a) and 6‐MV 3 × 3 cm^2^ beam (b).

The effect of introducing a slight tilt when films are placed in parallel orientation for the 220‐kVp and 6‐MV 3 × 3 cm^2^ beams is demonstrated in Fig. 6. Evidently, even a small tilt of 1° resulted in notably improved agreement between film and water phantom PDD. The mean absolute difference between the water and 1°‐tilt PDD decreased to 1.7% from 4.7% for 0°‐tilt in the 220‐kVp beam. The improvement for larger tilt angles was negligible. For the 6‐MV beam, the mean dose difference between water and film PDD for 1°, 2°, and 3°‐tilt was 0.8%, 0.5%, and 0.2%, respectively, which was an improvement over the 5% dose difference found with a 0°‐tilt.

### 3.E Experimental data

**Fig. 7:**
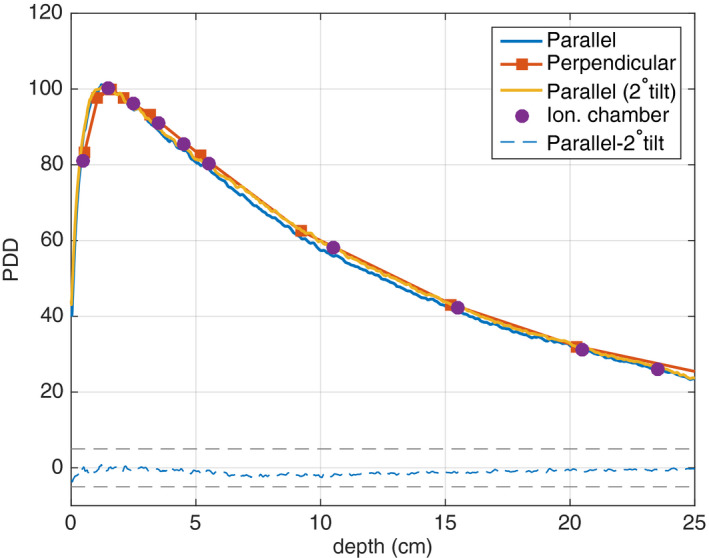
Experimental 6‐MV 3 × 3 cm^2^ beam PDD curve comparison for films placed in a solid water phantom in parallel and perpendicular orientation and with a 2°‐tilt with respect to the beam axis. Ionization chamber data as well as the difference for data acquired with film in parallel orientation and with the 2°‐tilt are also presented.

The results from the experimental measurements for a 6‐MV 3 × 3 cm^2^ beam performed with a TrueBeam linac for films placed in a solid water phantom are presented in Fig. 7. The trends of the MC simulation results persisted in the experimental data. The PDD data for films placed in perpendicular orientation closely matched ionization chamber measurements, the mean difference between the two measurements was 0.6%. The PDD values for film in parallel orientation were consistently 1–3% lower than the ionization chamber data, however, the data acquired with a 2° gantry tilt reduced the difference to 0.7%.

**Fig. 8:**
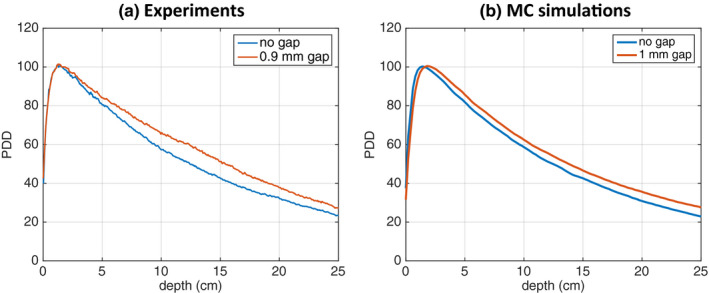
PDD curve comparison for film placed in a phantom in parallel orientation without an air gap and with an air gap irradiated with a 6‐MV 3 × 3 cm^2^ beam. Experimental data for a single 0.9‐mm air gap on one side of the film are shown in (a), MC simulation data with a 1‐mm air gap on either side of the film are shown in (b).

Experimental comparison of PDDs acquired with a film in parallel orientation with and without a 0.9‐mm air gap are presented in [Fig. 8(a)]. PDD values for the film with the air gap were consistently overestimated at depths beyond d_max_, by up to 10.0% at 13.4 cm. When compared to the ionization chamber data, the air gap PDD at 13.4‐cm depth was overestimated by 8.5%. For comparison, MC results for a divergent 6‐MV 3 × 3 cm^2^ beam with a 100‐cm SSD and 1‐mm air gaps on either side of the film are presented in [Fig. 8(b)]. While the MC model was by no means validated for our linac, the qualitative trend found in the measurement data persisted. PDD values for film with air gaps were also consistently overestimated, albeit to a lower degree, at depths beyond d_max_, by up to 4.8% at 19.8 cm.

## 4 Discussion

We have presented MC simulations of dose deposition in the active layer of EBT3 Gafchromic^TM^ films sandwiched in a water phantom and compared the resulting film PDDs with those scored in a homogeneous water phantom. Our MC simulations were ideal in nature: the films were assumed to be perfectly flat and geometrically uniform and we did not take into account the spectral changes of the beam that would affect the measurements of film OD and therefore absorbed dose. Additionally, only parallel beams without any beam divergence or beam penumbra were simulated. Some of the limitations of our study, partly alleviated by presentation of experimental data for a single beam energy and beam size, are discussed at the end of this section. Nonetheless, a number of observations can be made from this work.

**Fig. 9:**
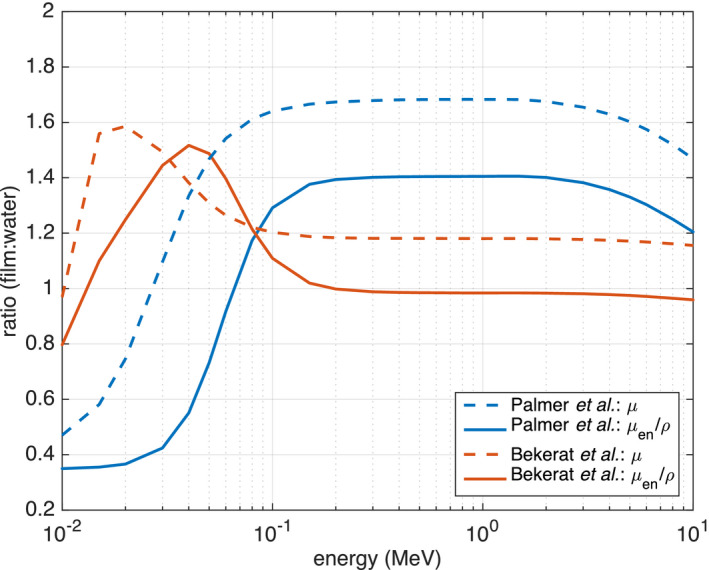
The ratio of the linear attenuation coefficient *µ* and the mass energy‐absorption coefficient *µ_en_/ρ* of the film active layer to water for the two different compositions of Gafchromic EBT3 films studied in this work. Data are derived from the NIST database.

First, it was demonstrated that dose calculated in EBT3 films oriented perpendicular to the beam axis more closely agreed with dose to water when compared to the films in parallel orientation (Fig. 3). The largest differences were found for the smallest 1 × 1 cm^2^ beam size for all beam energies. For example, when orthovoltage beam PDDs were normalized to 1‐cm depth, the 220‐kV photon beam suffered from the largest overall dose discrepancy of −12.9% at 6.2‐cm depth for films placed in parallel orientation. With the film in perpendicular orientation, however, the maximum dose discrepancy decreased to −3.3% at a depth of 7.0 cm. The 6‐MV beam simulations demonstrated a weaker dependence on film orientation; the maximum difference between the parallel film orientation and water PDD was −7.7% at 13.6‐cm depth and decreased to 1.4% at 2.0‐cm depth for the perpendicular film orientation. The 120‐kVp beam simulations showed that while a perpendicular film orientation stillresulted in more accurate dose measurements relative to the water phantom PDDs, the improvement relative to the parallel orientation would be the smallest of the three beam energies and increased with increasing field size. Altogether, the largest PDD differences between films in parallel orientation and the homogeneous water phantom were observed for the 220‐kVp beam. This can be explained by the large energy dependence for films in this beam (similar to the 120‐kVp beam) combined with the increased beam attenuation, relative to water, when compared to the 120‐kVp beam (Fig. 9). For energies lower than 60 keV, x‐ray beam attenuation in the film active layer is lower than beam attenuation in water, which will result in lower 120‐kVp beam attenuation compared to the 220‐kVp beam.

Second, it was demonstrated that the presence of air gaps on the side of film placed in parallel orientation altered the magnitude of the absorbed dose (Fig. 4). The dose discrepancy increased with increasing air gap and beam energy. Even for a small 0.10‐mm air gap, doses were underestimated by up to 4% in the buildup region for the 6‐MV 1 × 1 cm^2^ beam. For the largest studied 1.00‐mm air gap, the film dose was underestimated by 21.2% in the buildup region for the same beam. The other beam energies were less affected by the air gap; for example, using the 1.00‐mm air gap, doses were underestimated by up to 6% for the 120‐kVp and 220‐kVp beams. Note that the maximum differences between simulations with and without air gaps, for all PDDs normalized to their respective maximum dose, were found to be less than 3% beyond the buildup region for all beam energies and field sizes. For the 1.00‐mm air gap, the depth depth of maximum dose for the 6‐MV beam shifted from 1.6 to 2.0 cm for the 3 × 3 cm^2^ beam and from 1.6 to 2.2 cm for the 1 × 1 cm^2^ beam. The buildup region of the 6‐MV beam plays an interesting role in PDD evaluations using films in parallel orientation and the presence of an air gap. The d_max_ may shift to larger depths as secondary electrons generated in the adjacent water travel farther in air than in water/film and the most energetic electrons deposit their energy at greater depth in the film as a result. As the air gap widens, the electrons therein can travel farther while d_max_ increases , as seen in Fig. 4 as well in Fig. 8. Based on these results, and due to the possibility of air gaps in solid water phantoms, films placed in perpendicular orientation with respect to the beam axis should be used for beam output measurements of high‐energy photon beams.

The third study investigating different film compositions revealed that the elemental composition of the active and polyester base layers of EBT3 films can have a significant impact on film dose deposition (Fig. 5). PDD curves for simulations using the newer composition data published by Palmer et al. in 2015 resulted in larger dose discrepancies, relative to the water PDD, when compared to simulations using the 2014 composition data from Bekerat et al. PDD agreement was most improved for the 6‐MV 1 × 1 cm^2^ beam, for which the maximum difference from the water PDD decreased from −7.7% to −3.3%. Figure 9 presents the ratio of *µ_en_/ρ* of film active layer to water for the two investigated film compositions. It is clear that the ratio of *µ_en_/ρ* is more constant for the Bekerat et al. composition than that of Palmer et al., resulting in lower energy dependence. In the energy range between 1 keV and 20 MeV, the ratio of *µ_en_/ρ*for the Bekerat et al. composition is 0.992 ± 0.219 (1 standard deviation) and 0.951 ± 0.472 for the Palmer et al. composition. The lower doses measured with the EBT3 film in parallel orientation can likely be attributed to the increased attenuation within the film active layer compared to water, as indicated by the linear attenuation coefficient ratio of film active layer to water being consistently higher than 1.0 (Fig. 9).

Fourth, it was demonstrated that a slight film tilt improved the accuracy of central axis depth doses (Fig. 6). Interestingly, for the 220‐kVp beam the film PDD was overestimated relative to the water PDD even for the smallest 1°‐tilt and no further improvement for 2°‐ or 3°‐tilts was observed. For the 6‐MV beam, however, the dose accuracy was the maximized using a 3°‐tilt and did not improve further for a 4°‐tilt (data not shown). The improvement in PDD agreement for a film slightly tilted with respect to the beam axis can be attributed to the increased beam attenuation in the film active layer relative to water. When a slight film tilt *α* is introduced, the path length through the active layer is significantly decreased from the measurement depth to *d_al_*/sin*(α)*, where *d_al_* is the thickness of active layer of 28 µm. For example, for a film tilt of 2º the path length reduces from the measurement depth to only 0.8 mm.

While most of the presented results are based on MC simulations, experimental data acquired for a 6‐MV 3 × 3 cm^2^ beam and presented in Fig. 7 and Fig. 8 qualitatively support the results of MC simulations. The PDD curve for a 0.9‐mm air gap in [Fig. 8(a)] shows the largest difference between measurements with no air gap at depths between 10 and 15 cm, suggesting that the air gap between the film and the solid water was likely inconsistent and largest at these depths.

For films in parallel orientation, the dose deposition is affected by the increased attenuation of the beam within the active layer compared to water, as well as different lateral scatter contributions from the adjacent polyester layer. Central axis PDDs are more sensitive to lateral scattering contributions as field size increases. For phantoms with films in perpendicular orientation, the attenuation difference in the thin active layer as well as in the polyester layer has a smaller effect on the PDD than for phantoms with films in parallel orientation. In the case where air gaps are introduced for films in parallel orientation, the lateral scatter from air contributes less to the central axis dose than would be the case for water.

As mentioned above, our study design did not include the response of EBT3 Gafchromic^TM^ films as a function of beam energy, which changes as the beam travels through the phantom. Thankfully EBT3 film has been shown to be less energy dependent than its previous generations.^14^, ^33^, ^34^, ^35^ Including the film energy response in our investigation would likely have minor implications on our findings for the 6‐MV photon beam, which is supported by the experimental results presented in Fig. 7 and Fig. 8. On the other hand, the changing film energy response at depth should be included in a future model for the low‐energy kilovoltage and orthovoltage beams.

Investigations of radiochromic film response using EGSnrc have been performed in the past. For example, Sutherland et al. investigated the energy response of EBT and EBT2 films^36^ and Bekerat et al. studied the energy response of EBT3 films.^27^ Experimentally, the response of Gafchromic^TM^ EBT2 film was studied by Arjomandy et al.^19^ The authors evaluated PDDs measured with films parallel to the beam axis with a 2°‐tilt and found good agreement with ionization chamber measurements over a wide range of beam energies and modalities.

Suchowerska et al. in 1999 investigated the effect of Kodak X Omat‐V radiographic film orientation on the evaluation of PDD^24^. This silver‐based film placed in parallel orientation resulted in up to 15% and 14% overestimation of dose at 25‐cm depth for a ^60^Co and a 6‐MV beam, respectively, compared to the perpendicular orientation which had agreed with ionization chamber measurements. The overestimation of dose for films in parallel orientation was explained by the increased film‐to‐water ratio of *µ_en_/ρ* at lower energies, raising rapidly for energies below 550 keV, exceeding a factor of 2 at ~ 150 keV. EBT3 films, on the other hand, are more tissue equivalent and the *µ_en_/ρ* ratio of film to water decreases by less than 1.5% from 2 MeV to 150 keV (Fig. 9). The lower absorbed doses observed in this tudy for EBT3 film in parallel orientation can be explained by the increased beam attenuation in the film active layer compared to water.

Due to the presence of the film in the perpendicular orientation, the effective depth of each film measurement is increased by approximately 161 µm and 66 µm per film for the Palmer et al. and Bekerat et al. compositions, respectively, in a 2‐MeV beam representing the mean energy of the 6‐MV source. For the other two beams, the difference between the actual film depth and the effective film depths was decreased, and thus the film depths were considered to be equal to the actual film depths in this study.

Based on our MC study, we present a short list of recommendations for PDD measurements with Gafchromic^TM^ EBT3 films.

1. If possible, use a number of films in perpendicular orientation with respect to beam axis. While dose measurement accuracy for high‐energy photon beams could be within 2%, dose measurement accuracy for kilovoltage and orthovoltage beams might be only 4–5%.
2. If a parallel film orientation is used, tilt the film at a small ~ 2° angle. This should improve dose measurement accuracy, especially for high‐energy photon beams. The dose at depth for kilovoltage and orthovoltage beams might be overestimated by ~ 3%.
3. Ensure that there are no air gaps between the film and the phantom. This is most critical for dose measurement accuracy in high‐energy photon beams and using small field sizes. Air gaps might result in an underestimation of absolute dose as well as a shift in d_max_.

## 5 Conclusion

We have presented a Monte Carlo study that highlighted some challenges with EBT3 film dosimetry for PDD evaluation when films are used in parallel orientation with respect to the beam axis. PDD differences between water and film dose distributions existed for all studied beam sizes and photon beam energies spanning from kilovoltage to megavoltage beams. By means of extensive computer simulations and limited experimental measurements, we have demonstrated that a slight ~ 2° film/beam tilt improves the accuracy of PDD evaluation and that air gaps cause overestimation of PDDs at depths beyond the buildup region. While films orientated parallel with the beam axis offer a convenient way to measure PDDs, we recommend that caution be taken when irradiating films in parallel orientation, namely ensuring that air gaps between the film and the phantom are minimized and that the film and beam axis are slightly tilted with respect to each other.

## Conflict of Interest

The authors have no conflict of interest.

## References

[1] Devic S . Radiochromic film dosimetry: past, present, and future. Physica Med. 2011;27:122–134. 10.1016/j.ejmp.2010.10.001 21050785

[2] Wilcox EE , Daskalov GM . Evaluation of GAFCHROMIC® EBT film for CyberKnife® dosimetry. Med Phys. 2007;34:1967–1974. 17654899 10.1118/1.2734384

[3] Polednik M , Madyan YA , Schneider F , et al. Evaluation of calculation algorithms implemented in different commercial planning systems on an anthropomorphic breast phantom using film dosimetry. Strahlenther Onkol. 2007;183:667–672. 18040610 10.1007/s00066-007-1775-1

[4] Borca VC , Pasquino M , Russo G , et al. Dosimetric characterization and use of GAFCHROMIC EBT3 film for IMRT dose verification. J Appl Clin Med Phys. 2013;14:158–171. 10.1120/jacmp.v14i2.4111 PMC5714357 23470940

[5] García‐Garduño OA , Celis MÁ , Lárraga‐Gutiérrez JM , Moreno‐Jiménez S , Martínez‐Dávalos A , Rodríguez‐Villafuerte M . Radiation transmission, leakage and beam penumbra measurements of a micro‐multileaf collimator using GafChromic EBT film. J Appl Clin Med Phys. 2008;9:90–98. 18716595 10.1120/jacmp.v9i3.2802 PMC5722293

[6] Crosbie J , Svalbe I , Midgley SM , Yagi N , Rogers PW , Lewis R . A method of dosimetry for synchrotron microbeam radiation therapy using radiochromic films of different sensitivity. Phys Med Biol. 2008;53:6861. 19001701 10.1088/0031-9155/53/23/014

[7] Su F‐C , Shi C , Papanikolaou N . Clinical application of GAFCHROMIC® EBT film for in vivo dose measurements of total body irradiation radiotherapy. Appl Radiat Isot. 2008;66:389–394. 18023587 10.1016/j.apradiso.2007.09.015

[8] Bufacchi A , Carosi A , Adorante N , et al. In vivo EBT radiochromic film dosimetry of electron beam for Total Skin Electron Therapy (TSET). Physica Med. 2007;23:67–72. 10.1016/j.ejmp.2007.03.003 17568545

[9] Zhao L , Das IJ . Gafchromic EBT film dosimetry in proton beams. Phys Med Biol. 2010;55:N291. 20427858 10.1088/0031-9155/55/10/N04

[10] Su F‐C , Liu Y , Stathakis S , Shi C , Esquivel C , Papanikolaou N . Dosimetry characteristics of GAFCHROMIC® EBT film responding to therapeutic electron beams. Appl Radiat Isot. 2007;65:1187–1192. 17590344 10.1016/j.apradiso.2007.05.005

[11] Darafsheh A , Hao Y , Maraghechi B , Cammin J , Reynoso FJ , Khan R . Influence of 0.35 T magnetic field on the response of EBT3 and EBT‐XD radiochromic films. Med Phys. 2020;47:4543–4552. 32502280 10.1002/mp.14313

[12] Rampado O , Garelli E , Ropolo R . Computed tomography dose measurements with radiochromic films and a flatbed scanner. Med Phys. 2010;37:189–196. 20175481 10.1118/1.3271584

[13] Gotanda R , Katsuda T , Gotanda T , Tabuchi A , Yatake H , Takeda Y . Dose distribution in pediatric CT head examination using a new phantom with radiochromic film. Australasian Phys Engineer Sci Med. 2008;31:339–344. 10.1007/BF03178604 19239061

[14] Hammer CG , Rosen BS , Fagerstrom JM , Culberson WS , DeWerd LA . Experimental investigation of GafChromic® EBT3 intrinsic energy dependence with kilovoltage x rays, 137Cs, and 60Co. Med Phys. 2018;45:448–459. 29159807 10.1002/mp.12682

[15] Jaccard M , Durán MT , Petersson K , et al. High dose‐per‐pulse electron beam dosimetry: commissioning of the Oriatron eRT6 prototype linear accelerator for preclinical use. Med Phys. 2018;45:863–874. 29206287 10.1002/mp.12713

[16] Bazalova M , Nelson G , Noll JM , Graves EE . Modality comparison for small animal radiotherapy: a simulation study. Med Phys. 2014;41:011710. 24387502 10.1118/1.4842415 PMC3888460

[17] Bazalova‐Carter M , Liu M , Palma B , et al. Comparison of film measurements and Monte Carlo simulations of dose delivered with very high‐energy electron beams in a polystyrene phantom. Med Phys. 2015;42:1606–1613. 25832051 10.1118/1.4914371

[18] Johnstone CD , Bazalova‐Carter M . MicroCT imaging dose to mouse organs using a validated Monte Carlo model of the small animal radiation research platform (SARRP). Phys Med Biol. 2018;63:115012. 29741161 10.1088/1361-6560/aac335

[19] Arjomandy B , Tailor R , Zhao L , Devic S . EBT2 film as a depth‐dose measurement tool for radiotherapy beams over a wide range of energies and modalities. Med Phys. 2012;39:912–921. 22320801 10.1118/1.3678989

[20] Esplen N , Therriault‐Proulx F , Beaulieu L , Bazalova‐Carter M . Preclinical dose verification using a 3D printed mouse phantom for radiobiology experiments. Med Phys. 2019;46:5249–5303. 31461781 10.1002/mp.13790

[21] Fontanarosa D , Orlandini LC , Andriani I , Bernardi L . Commissioning Varian enhanced dynamic wedge in the PINNACLE treatment planning system using Gafchromic™ EBT film. Med Phys. 2009;36:4504–4510. 19928081 10.1118/1.3223621

[22] Dutreix J , Dutreix A . Film dosimetry of high‐energy electrons. Ann N Y Acad Sci. 1969;161:33–43. 4979829 10.1111/j.1749-6632.1969.tb34039.x

[23] Williamson JF , Khan FM , Sharma SC . Film dosimetry of megavoltage photon beams: a practical method of isodensity‐to‐isodose curve conversion. Med Phys. 1981;8:94–98. 7207433 10.1118/1.594913

[24] Suchowerska N , Hoban P , Davison A , Metcalfe P . Perturbation of radiotherapy beams by radiographic film: measurements and Monte Carlo simulations. Phys Med Biol. 1999;44:1755. 10442711 10.1088/0031-9155/44/7/314

[25] Suchowerska N , Hoban P , Butson M , Davison A , Metcalfe P . Directional dependence in film dosimetry: radiographic and radiochromic film. Phys Med Biol. 2001;46:1391. 11384060 10.1088/0031-9155/46/5/305

[26] Palmer AL , Dimitriadis A , Nisbet A , Clark CH . Evaluation of Gafchromic EBT‐XD film, with comparison to EBT3 film, and application in high dose radiotherapy verification. Phys Med Biol. 2015;60:8741–8752. 26512917 10.1088/0031-9155/60/22/8741

[27] Bekerat H , Devic S , DeBlois F , et al. Improving the energy response of external beam therapy (EBT) GafChromicTM dosimetry films at low energies (≤ 100 keV). Med Phys. 2014;41:022101. 24506633 10.1118/1.4860157

[28] Walters BRB , Kawrakow I , Rogers DWO . DOSXYZnrc users manual. In: NRCC; 2007.

[29] Bazalova M , Zhou H , Keall PJ , Graves EE . Kilovoltage beam Monte Carlo dose calculations in submillimeter voxels for small animal radiotherapy. Med Phys. 2009;36:4991–4999. 19994508 10.1118/1.3238465 PMC2773455

[30] Mohan R , Chui C , Lidofsky L . Energy and angular distributions of photons from medical linear accelerators. Med Phys. 1985;12:592–597. 4046993 10.1118/1.595680

[31] Gafchromic™ dosimetry media, type EBT3 2017 www.gafchromic.com/documents/EBT3_Specifications.pdf Accessed 2020.

[32] Niroomand‐Rad A , Blackwell CR , Coursey BM , et al. Radiochromic film dosimetry: recommendations of AAPM radiation therapy committee task group 55. Med Phys. 1998;25:2093–2115. 9829234 10.1118/1.598407

[33] Dreindl R , Georg D , Stock M . Radiochromic film dosimetry: Considerations on precision and accuracy for EBT2 and EBT3 type films. Zeitschrift für Medizinische Physik. 2014;24:153–163. 24055395 10.1016/j.zemedi.2013.08.002

[34] Sorriaux J , Kacperek A , Rossomme S , et al. Evaluation of Gafchromic® EBT3 films characteristics in therapy photon, electron and proton beams. Physica Med. 2013;29:599–606. 10.1016/j.ejmp.2012.10.001 23107430

[35] León‐Marroquín EY , Mulrow DJ , Khan R , Darafsheh A . Spectral analysis of the EBT3 radiochromic films for clinical photon and electron beams. Med Phys. 2019;46:973–982. 30536832 10.1002/mp.13330

[36] Sutherland JGH , Rogers DWO . Monte Carlo calculated absorbed‐dose energy dependence of EBT and EBT2 film. Med Phys. 2010;37:1110–1116. 20384246 10.1118/1.3301574 PMC2837726

